# Percutaneous transhepatic placement of a stent-graft to treat a delayed mesoportal hemorrhage after pancreaticoduodenectomy

**DOI:** 10.1186/1477-7819-12-315

**Published:** 2014-10-15

**Authors:** Michael Ginsburg, Hector Ferral, Marc J Alonzo, Mark S Talamonti

**Affiliations:** Department of Radiology, Stanford Hospital & Clinics, 300 Pasteur Drive, Stanford, CA 94305 USA; Department of Radiology, NorthShore University HealthSystem, 2650 Ridge Avenue, Evanston, Illinois 60201 USA; Department of Surgery, NorthShore University HealthSystem, 2650 Ridge Avenue, Evanston, Illinois 60201 USA

## Abstract

Postoperative hemorrhage is one of the most severe complications after pancreaticoduodenectomy. While detection of bleeding from adjacent arteries via conventional angiography and treatment with endovascular arterial coil embolization has been well established, to date no reports of percutaneous therapy for mesoportal hemorrhage have been published. This article describes an unusual case of delayed post-pancreaticoduodenectomy hemorrhage detected on a fluoroscopic drain check and treated with percutaneous transhepatic covered stent placement.

## Background

Post-pancreaticoduodenectomy hemorrhage represents a rare, but one of the most serious complications in pancreatic surgery associated with high mortality rate [1, 2]. This dreaded complication is often preceded by sentinel bleed and tends to occur as a result of postoperative pancreatic fistula causing vascular erosion and bleeding [1–5]. While arterial embolization has been shown to be an effective first line treatment in delayed post-pancreaticoduodenectomy hemorrhage (DPH) in multiple studies, up to 50% of patients require a second laparotomy for non-localized bleeding site [2–4]. We report a case of DPH in a patient with post-operative course complicated by pancreatic fistula that presented with a sentinel bleed. The mesenteric angiogram was negative and hemorrhage source was incidentally detected on a fluoroscopic drain check and was subsequently treated with percutaneous transhepatic covered stent placement.

## Case presentation

**Figure 1:**
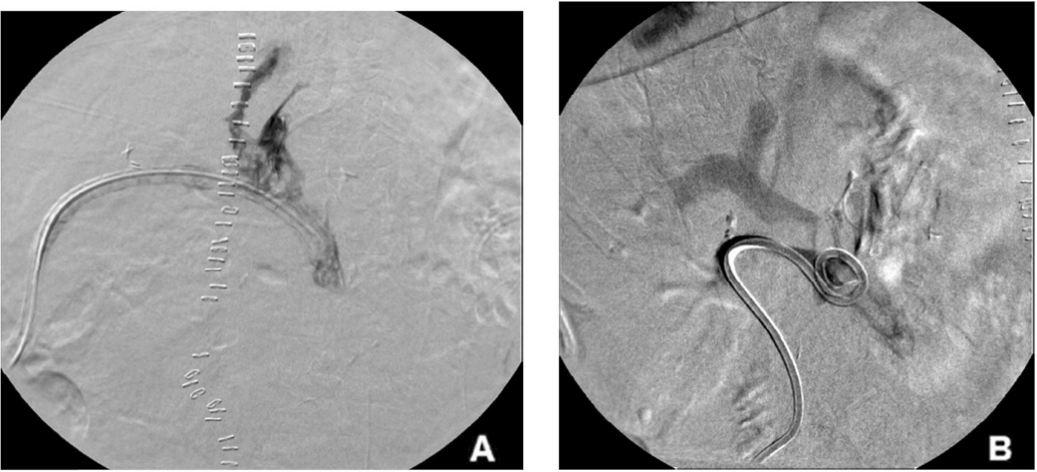
**Fluoroscopic drain contrast injection. (A)** Jackson-Pratt (JP) surgical drain injection showing expected opacification of the postoperative bed with no evidence of fistula. **(B)** Post-exchange of JP drain for an all**-**purpose drainage catheter; opacification of the portal vein at the superior mesenteric vein and portal vein confluence is observed after all-purpose drainage catheter injection.

**Figure 2:**
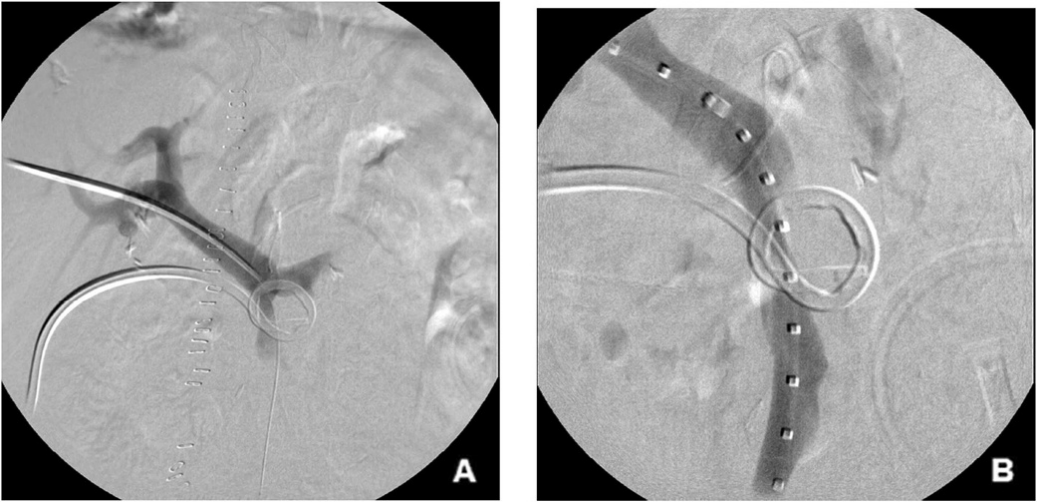
**Percutaneous transhepatic portogram (A) Percutaneous transhepatic portogram showing 5-Fr vascular sheath within the main portal vein with no evidence of active extravasation.** Slight narrowing of the superior mesenteric vein (SMV) at its confluence with the main portal vein is noted. **(B)** A 7-Fr vascular sheath within the main portal vein and calibrated sizing catheter in the SMV demonstrating wall contour irregularity and narrowing of the SMV at the site of suspected bleeding based on the drain check.

**Figure 3:**
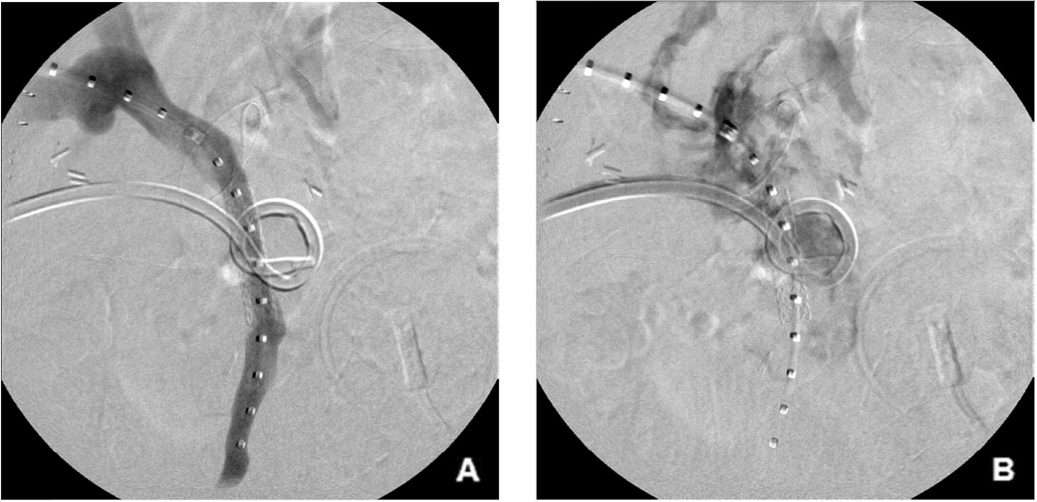
**Post stent placement percutaneous transhepatic portogram and fluoroscopic drain contrast injection (A** **) Post-stent deployment portogram shows covered balloon expandable iCAST stent in superior mesenteric vein (SMV) with interval resolution of vessel wall contour irregularity and narrowing. (B)** A 12-Fr all-purpose drainage catheter injection confirming successful seal of the fistula between the SMV and the postoperative cavity.

The patient is a 77-year-old female who underwent pancreaticoduodenectomy for adenocarcinoma of the ampulla of Vater. Tumor adherence to the superior mesenteric-portal vein confluence was identified during surgery and required a lateral wall resection with primary repair to achieve complete tumor extirpation. The post-surgical inpatient course was complicated by the development of a pancreatic fistula, which was properly controlled by a Jackson-Pratt (JP) drain placed during surgery. The patient presented with bleeding on postoperative day 18 with a sudden onset frank bloody output (415 cm^3^/24 hours) from her JP drain consistent with a sentinel bleed. Hemoglobin levels dropped from 9.8 to 7.8 g/dl in a period of 24 hours. She remained hemodynamically stable and was initially managed by transfusion of one unit of packed red blood cells and intravenous fluids.A mesenteric angiogram with possible embolization was requested, along with a JP drain check and exchange. Selective catheterization and angiography of the celiac and superior mesenteric arteries demonstrated no evidence for arterial source of bleeding or pseudoaneurysm. The mesenteric angiogram was carried to the venous phase and no active bleeding was detected. The JP drain was then injected with a diluted nonionic iodinated contrast agent (Figure 1A). This showed expected opacification of the postoperative bed and no evidence of fistulous communication to the bowel. The JP was then exchanged over wire for a 12-Fr all-purpose drainage catheter (Flexima APD, Boston Scientific, Natick, MA, USA) and contrast was injected again to confirm a good catheter position. This catheter injection demonstrated opacification of the portal vein (Figure 1B).Given the findings, it was decided to proceed with percutaneous transhepatic stent placement. A percutaneous transhepatic puncture of a right portal venous branch was performed with a 22-gauge needle (Neff Percutaneous Access Set; Cook Medical, Bloomington, IN, USA) under sonographic and fluoroscopic guidance. The needle was exchanged for a 4-Fr coaxial dilator followed by placement of a 7-Fr vascular sheath (Boston Scientific) over a 0.035-inch Amplatz guidewire (Boston Scientific). Direct main portography demonstrated no evidence of active extravasation; however, a subtle narrowing area and wall irregularity of the superior mesenteric vein (SMV) at its confluence with the main portal vein was noted (Figure 2A) corresponding to the origin of portal vein opacification, and the site of suspected bleeding, based on the drain check (Figure 2B). A direct portogram was then performed using a 5-Fr calibrated sizing catheter to determine the optimal size and location for the planned endovascular stent graft placement. A polytetrafluoroethylene-covered balloon expandable iCAST stent (Atrium Medical Corporation, Hudson, NH, USA) was deployed under fluoroscopic guidance into the SMV (stent diameter 8 mm, length 38 mm) to seal the suspected bleeding site (Figure 3A). The 12-Fr all-purpose drainage catheter was then again injected to confirm successful seal of the portal vein and SMV junction fistula with the postoperative cavity (Figure 3B). The percutaneous transhepatic tract was embolized using a single 4 mm × 14 cm Nester coil (Cook Medical). Patient bleeding resolved and the patient was discharged home on postoperative day 32 and 2 weeks after percutaneous stent-graft placement.

Contrast-enhanced computed tomography 7 months after SMV stent-graft placement demonstrated a widely patent stent-graft and no residual fluid collection. The pancreatic fistula resolved within 4 weeks after stent placement and final abscessogram and drain removal was performed 4 weeks after stenting. The patient was seen again in clinic 7 months after intervention and was asymptomatic, recovering well, with no further bleeding episodes.

## Discussion

Delayed post-pancreaticoduodenectomy hemorrhage (DPH) is a life threatening complication seen in less than 5% of patients, but associated with a mortality rate as high as 60% [1, 2]. The international consensus classification established by the International Study Group for Pancreatic Surgery categorize post-pancreatectomy hemorrhage based on timing, severity and site of bleeding [1]. While early hemorrhage, defined as bleeding within the first 24 hours after surgery, is most commonly a result of surgical technical failure, delayed hemorrhage, defined as any bleeding after 24 hours and often days to weeks later, is thought to be caused by erosion of skeletonized visceral vessels as a result of postoperative pancreatic fistula or abscess [1, 3]. The term “sentinel bleed” refers to an isolated episode of bleeding, usually from an abdominal drain, implying structural vascular defect and requiring immediate evaluation due to possible impending major hemorrhage [3–5].

Multiple studies have advocated arterial embolization as a primary intervention for extraluminal DPH with the rationale of avoiding significant morbidity and mortality associated with a technically difficult reoperation [3–6]. The reported overall success rate for angiographic hemostasis of extraluminal DPH has ranged between 50% and 80% [4–6]. The postulated causes of false-negative angiographies include the intermittent character of bleeding episodes and venous bleeding, which is difficult to identify after an arterial injection [6]. To the best of our knowledge this is the first described detection of mesoportal venous bleeding via drain check as well as percutaneous transhepatic treatment of DPH due to a bleeding SMV.

Insertion of self-expandable stents in the portal vein or SMV has been used only rarely; however, the few reports in the literature are encouraging. Hellman and colleagues [7] used self-expandable stents through a stenotic SMV caused by midgut carcinoid disease, with four out of seven patients experiencing improvement of abdominal symptoms and one bleeding complication related to liver puncture that required a second intervention. Percutaneous transhepatic portal vein stent placement was deemed to be a safe and effective treatment for portal vein stenosis caused by both a benign entity or by tumor recurrence after curative surgery for pancreatic or biliary neoplasm [8–10]. However, Kim and collegaues reported three major complications (septicemia, liver abscess, and acute portal venous thrombosis) out of a total of 18 patients after successful stent placement [10].

In our case, the stent was placed successfully without procedural complications, resolving the patient’s DPH and avoiding reoperation. While venous bleeding is a rare cause of DPH, with the exact incidence unknown, it is important to consider it when the mesenteric angiogram is negative. In retrospect, it can be argued that triple-phase computed tomography should have been performed as it could have showed the cause, approximate site and nature of the bleeding [3]. However, it would not have eliminated the need for a mesenteric angiogram and likely would not have been able to identify the exact site of the venous bleeding.

## Conclusion

In conclusion, DPH is an important and potentially lethal complication. When the mesenteric angiogram is negative, a venous source for the bleed should be considered and investigated. Our experience shows that endovascular treatment with percutaneous transhepatic stenting is safe and may be an effective option in management of post-surgical mesenteric venous bleeding.

## Consent

Written informed consent was obtained from the patient for publication of this Case report and any accompanying images. A copy of the written consent is available for review by the Editor-in-Chief of this journal.

## Abbreviations

DPH: Delayed post-pancreaticoduodenectomy hemorrhage
JP: Jackson-Pratt
SMV: Superior mesenteric vein.
